# Geriatrician-led Interdisciplinary Rounds to decrease Hospital readmissions

**DOI:** 10.1093/geroni/igaf122.2190

**Published:** 2025-12-31

**Authors:** Julio Defillo Draiby, Michael Capilupi, Ka Ly, Barbara Mckenzie, Wen-Chih Wu

**Affiliations:** VA Providence Healthcare System, Palm City, Rhode Island, United States; Brown University, Providence, Rhode Island, United States; VA Providence Healthcare System, Providence, Rhode Island, United States; VA Providence Health Care System, Providence, Rhode Island, United States; VA Providence Healthcare System, Providence, Rhode Island, United States

## Abstract

**Background:**

Age-Friendly Health Care Systems (AFHS) framework is used as a way to care for Older Adults¹. Older patients occupy nearly half of the inpatient beds in community hospitals². We wanted to determine if a Geriatrician-led interdisciplinary team was associated with changes in key performance indicators.

**Methods:**

Veterans 75 years or older assigned to a ten-bed virtual capacity, inpatient medical ward evaluated by a Geriatric led interdisciplinary (GEC) team utilizing the mentation, mobility, what matters, medications framework (4Ms) from the AFHS, generating a Geriatric Assessment note. Patients were excluded if admitted with a decompensated psychiatric disease or needed intensive or step-down level of care. After the initial evaluation, the GEC discussed daily until discharge. A total of 233 Veterans admitted to the AFHS was compared against 418 age-matched Veterans not admitted to AFHS (non-AFHS) during the same period (10/1/2023 – 6/30/2024; n = 651 total) on their length-of-stay (LOS), and 30-day readmission and mortality rates using multivariate linear and logistic regression where applicable adjusted for Charlson comorbidity and care assessment needs (CAN) scores.

**Results:**

Veterans admitted to the AFHS had a similar age but significantly higher Charlson and CAN scores. LOS, 30-day readmission, and mortality rates were similar between the two groups. Qualitative interviews of both patients and caregivers were also positive.

**Conclusion:**

In this Geriatric led AFHS improvement teamwork, we found that it had similar rates of mortality and readmissions in older veterans admitted to the medical ward despite the AFHS having a sicker population.

